# The Relationship between Early Growth and Survival of Hatchling Saltwater Crocodiles (*Crocodylus porosus*) in Captivity

**DOI:** 10.1371/journal.pone.0100276

**Published:** 2014-06-24

**Authors:** Matthew L. Brien, Grahame J. Webb, Keith McGuinness, Keith A. Christian

**Affiliations:** 1 Research Institute for the Environment and Livelihoods, Charles Darwin University, Darwin, NT, Australia; 2 Wildlife Management International Pty. Limited, Karama, NT, Australia; Monash University, Australia

## Abstract

Hatchling fitness in crocodilians is affected by “runtism” or failure to thrive syndrome (FTT) in captivity. In this study, 300 hatchling *C. porosus*, artificially incubated at 32°C for most of their embryonic development, were raised in semi-controlled conditions, with growth criteria derived for the early detection of FTT (within 24 days). Body mass, four days after hatching (BM_4d_), was correlated with egg size and was highly clutch specific, while snout-vent length (SVL_4d_) was much more variable within and between clutches. For the majority of hatchlings growth trajectories within the first 24 days continued to 90 days and could be used to predict FTT affliction up to 300 days, highlighting the importance of early growth. Growth and survival of hatchling *C. porosus* in captivity was not influenced by initial size (BM_4d_), with a slight tendency for smaller hatchlings to grow faster in the immediate post-hatching period. Strong clutch effects (12 clutches) on affliction with FTT were apparent, but could not be explained by measured clutch variables or other factors. Among individuals not afflicted by FTT (N = 245), mean growth was highly clutch specific, and the variation could be explained by an interaction between clutch and season. FTT affliction was 2.5 times higher among clutches (N = 7) that hatched later in the year when mean minimum air temperatures were lower, compared with those clutches (N = 5) that hatched early in the year. The results of this study highlight the importance of early growth in hatchling *C. porosus*, which has implications for the captive management of this species.

## Introduction

Initial offspring size in the wild and in captivity can be expected to confer short to long term fitness advantages if it improves the ability to forage or capture food, avoid predation, compete with conspecifics, survive adverse environmental conditions [1] [2] [3] [4] [5] and ultimately produce more offspring [6] [5]. This has been demonstrated in a wide range of mammals [7] [8], birds [9] [10], reptiles [11] [12], amphibians [13] [14], fish [15] [16], and arthropods [17] [18] [4].

Large variation in offspring size and early growth rates are common within and between species [2] [5], between different populations of the same species [14] [19] [3], and between siblings from the same clutch [5]. Maternal size and condition [20] [21], genetic effects [22], multiple paternity, and conditions experienced prior to and after birth or hatching [22] may all be involved. With several species of mammals [23], snakes [22], fish [24], and frogs [25], the early nutritional environment is reportedly just as important as genetic influences in creating irreversible changes in growth rate and survival which affect long-term fitness [26] [27] [28] [22].

Despite larger offspring size often being correlated with higher rates of initial growth and survival (‘bigger is better’) [1] [16] [3] [22] [29], there are many exceptions. There can be small or no effects of initial size on fitness [30] [2] [31], skewed effects in which intermediate sized individuals are the most fit [32], or negative effects in which initial high growth rates are detrimental to fitness [33] [34]. Among reptiles, the ‘bigger is better’ hypothesis appears to be generally supported [1] [35] [12] [22], but there are few data available for crocodilians in the wild or in captivity.

This study examines growth and survival in captive raised hatchling saltwater crocodiles (*Crocodylus porosus*), mostly from wild collected eggs (10 of 12 clutches). Average clutch size for *C. porosus* in Australia is around 50 eggs (range 2–78; 65.4 to 147.0 g eggs) producing hatchlings from 41.4 to 93.6 g [36] [37]. In the wild an estimated 54% of hatchlings survive to 1-year-of-age [36] [38], whereas in captivity survival rates are higher, but vary greatly between establishments using different raising techniques [39]. The primary cause of mortality in captivity (49% of all hatchling deaths in captivity) is ‘runtism’ [40] [41] [39] [42], which is in essence a failure to thrive syndrome (FTT) involving voluntary starvation, which reduces immunity to disease and causes death between 70 and 200 days post-hatching [40] [41] [39], with time to death dependent upon initial weight [43]. The root causes of FTT in hatchlings remain poorly understood, but genetic [39] and incubation effects [37], elevated corticosterone levels [44], agonistic social interactions [45], various aspects of the raising environment (temperature, noise, visual stimuli) and management protocols (density, size disparity, disturbance) have all been implicated [46] [40] [41] [39].

The central aims of the present study were to determine whether growth trajectories established within the first three weeks post-hatching could be used as indices of short-term fitness. The degree to which size and body condition at hatching influences post-hatching growth is examined. Particular attention is focussed on clutch effects on both growth rates and the incidence of FTT. The FTT phenomenon in *C. porosus* and its implications on short-term fitness in captivity and in the wild are discussed.

## Materials and Methods

This project was conducted under the approval of the Animal Ethics Committee of Charles Darwin University (permit no. A11003).

### Clutches, eggs and incubation

Saltwater crocodile eggs and hatchlings used in these experiments were provided by Wildlife Management International (WMI; Darwin, Australia). There were effectively two groups of hatchlings, those clutches that hatched early in the year (16–24 January 2011; 5 clutches; N = 120) when ambient conditions were warmer, and those that hatched later in the year (29 March-22 May 2011; 7 clutches; N = 180) when conditions were cooler. Eggs came from wild nests (N = 10) collected 1–50 days after laying and captive nests (N = 2), collected 1–2 days after laying. Egg temperature within the nest (T_nest_) was measured at the time of collection with calibrated thermometers, 2–3 eggs deep in the clutch. Daily fluctuations in T_nest_ are reasonably modest but peak at 19:00 h to 21:00 h [47]. Measured T_nest_ and the time of measurement were used as a clutch-specific index for aligning what the mean (T_n.mean_) and maximum (T_n.max_) nest temperatures may have been up to the time of collection. Variation in egg size within clutches of *C. porosus* is low [38], and so mean egg size (mass, length, width) was measured from only 10 eggs per clutch. All eggs are carried within the oviducts of females prior to laying, and thus total clutch mass or volume is the best clutch-specific indicator of female size [38]. The age of each clutch at the time of collection and the number of infertile eggs and eggs with dead embryos were estimated using methods described previously [36] [49].

Incubation to hatching was completed for all eggs at constant 32°C (±0.2°C) and 98–100% humidity, which produces hatchlings with the highest rates of growth and survival [37]. Eggs were inspected regularly and the embryos of any dead eggs were used to determine whether death had occurred during incubation or prior to collection. Hatching typically occurred on the same day for each clutch. Hatchlings with deformities or which appeared to have excessive abdominal yolk, often resulting in lower survivorship, were excluded from the experiment. Sex was not determined, but 32°C is a male producing temperature, and the sensitive period for sex determination for the majority of eggs (10 of 12 clutches) occurred in the incubator [49] [50] [51]. For the two oldest clutches, sex may have been determined in the field. One (T_n.mean_  = 28.6°C at 50 days) was probably 100% female, whereas the other (T_n.mean_  = 33.3°C at 36 days) may have contained males and high temperature females [49] [50] [51] [37]. All hatchings were held in the incubator (32°C) in crates for three days after hatching before release into their raising enclosures (day 4) and being fed.

### Experimental enclosures

Two types of experimental enclosures (initial and final) were used. Hatchlings were housed between days 4 and 24 in sibling-only groups of 7–10 individuals in the initial enclosures. They were box shaped (170×100×50 cm high) fibreglass enclosures with a land area (70×100 cm) that gradually sloped down to a water area (100×100 cm; ≤8 cm deep). At 24 days, hatchlings were transferred into the final enclosures, in mixed clutch groups of twenty individual hatchlings of similar size. The final enclosures were 3 m^2^ box-shaped concrete pens (150×200×150 cm high), with a land area (150×80 cm) that gradually sloped to a water area (150×120 cm; ≤19 cm deep). Each enclosure had a basking cage (100×120 cm) attached to the outside and accessible through an opening (20×10 cm) in the wall, which effectively increased the enclosure area from 3 to 4.2 m^2^. The cage increased the range of thermal options available to hatchlings. Hatchlings remained in the final enclosures up until a maximum of 10 months of age (300 days), but were sorted on the basis of size every 3–4 weeks [52] [53] [54]. Hence, density remained the same but the individuals in each final enclosure did not.

A “hide area” [52] [53] [54] was provided in all initial and final enclosures. Each was 80×90 cm, constructed of eight lengths (80 cm long) of 10 cm (diameter) PVC pipe strapped together in the horizontal plane and mounted on legs (5 cm). Hides were centrally positioned in the water (partly immersed) and overhung the land. One hide area was provided in the initial fibreglass enclosures, and two in each final enclosure. All hatchlings were subjected to a natural light cycle. Water temperature (T_w_) was maintained at 31–32°C with thermostatically controlled injection of warm water (initial enclosures) or submerged heating pipes (final enclosures). Air temperature (T_a_) averaged around 32–34°C but varied from 26–36°C at different times of the day depending on ambient temperatures. All animals were fed chopped red meat supplemented with di-calcium phosphate (4% by weight) and a multivitamin supplement (1%) at 16:00–17:00 h six days a week, with waste removed the following morning (08:00–09:00 h) when the water was changed. Equilibration of T_w_ after water changes took 0.5 to 1.5 hours.

### Identification and measurements

A single uniquely numbered metal webbing tag (Small animal tag 1005-3, National Band and Tag Co.) was attached to the rear back right foot at the time of hatching. Snout-vent length (SVL in mm to the anterior of the cloaca) and body mass (BM in g) were measured when the animals were introduced into the initial enclosures at 4 days of age, at 24 days of age when transferred to the final enclosures, and again at 70 to 194 days of age (depending on hatch date; Data S1). All hatchlings were fasted the day prior to measurements being taken. Fasting for 48 hours prior to measurement does not affect growth rates but longer periods of fasting do [43]. These data allowed a size-age curve to be constructed for each individual, from which, size at 90 days could be predicted, which avoided problems associated with the different real ages of individuals. These measurement intervals reflect previous indications that growth patterns established in the first few weeks and months are an important index of growth and survival after that time in crocodilians [55]. Hatchling *C. porosus* that are afflicted by FTT can be expected to succumb to mortality from 70–200 days post-hatching [39], so although measurements were not taken after 70–194 days, mortalities were recorded up 300 days (10 months) after which survival rates tend to be 95–97% [39].

### Statistical analyses

All statistical analyses were performed using JMP 8.0 statistical software [56]. Where appropriate, data were checked for normality (Shapiro-Wilk's test) and homoscedasticity (Cochran's test) prior to statistical analysis. Morphometric relationships between egg length (EL) and egg mass (EM) of each clutch with SVL_4d_ and BM_4d_; SVL, BM, and body condition (BC  =  BM/SVL g/mm) at 4 (SVL_4d_; BM_4d_), 24 (SVL_24d_; BM_24d_), and 90 (SVL_90d_; BM_90d_) days of age, and growth in BM between 4 and 24 days of age (G_BM4-24d_) and 24 and 90 days of age (G_BM24-90d_) were examined using regression analyses. Size at 90 days was predicted from the size-age relationship for each individual at 4, 24 and 70 to 194 days (dependent on actual age). As a check on biases associated with prediction, the actual BM_90d_ and SVL_90d_ of a sample of animals measured at 90 days (N = 48) were compared with the predicted values using paired *t*-tests. No significant difference was detected for the actual and predicted values of either BM_90d_ (*t* = 0.26; df  = 94; *P* = 0.79) or SVL_90d_ (*t* = 0.56; df  = 94; *P* = 0.58). We examined the effect of season (early: 16–24 January, N = 5; late: 29 March – 22 May, N = 7) on egg mass, hatchling size and growth (SVL and BM) at 4, 24, and 90 days, and %FTT using a PERMANOVA with clutch as a random factor nested within season. In PERMANOVA, probabilities that treatments are significantly different from each other are generated by permutation, which requires only limited assumptions about the distribution of the data: in particular, normality of the data is neither assumed nor required [57]. The analysis was conducted with 1000 permutations in the PERMANOVA+ add-in for PRIMER [57]. Regression analyses were also used to predict the probability of %FTT affliction up to 300 days from G_BM4-24d_ and BC_24d_, and BM_24d_, using progress means (N = 20 animals). Unequal *t*-tests were used to examine differences in BM_4d_ between hatchlings that became afflicted with FTT (N = 55) and those that survived (N = 245). A Pearson's chi-square test was used to examine the effect of clutch on the proportion of individuals that died from FTT affliction. The effect of clutch on size and growth of non FTT animals (N = 245) was analysed with an ANOVA. All means are reported ± one standard error with sample sizes.

## Results

### Clutch, incubation and hatchling characteristics

The wild and captive laid clutches had different numbers of different sized eggs, which produced different sized hatchlings and came from different sized and aged females (indicated by total clutch mass). Clutches were collected at different embryo ages from nests with different temperatures that were laid at different times. Clutches also had different rates of infertility and embryo mortality before and during incubation, ultimately producing different proportions of apparently normal hatchlings (Table 1). The raising experiments also occurred at different times of year, and despite T_w_ being constant, T_a_ varied with the prevailing ambient temperatures.

**Table 1:** Clutch-specific details associated with the hatchlings used in the raising trials and their incubation.

	Clutch ID												
Characteristic	A20	A44	A70	A71	A75	A77	BP4	CB5	M28	M43	M55	M62	Grand means ±SD
Age at collection	1	4	24	12	1	1	0	0	16	24	46	42	12.9±15.6
Lay day of year	10	82	125	132	141	141	22	22	15	6	91	114	75.1±56.0
Actual T_n_ (°C)	29.8	29.8	27.2	28.4	31.9	27.2	30.7	29.5	32.4	32.9	33.2	28	30.1±2.2
T_n.mean_ (°C)	29.9	31	28.2	29.2	32.2	27.5	31.5	31.4	32.7	33	33.3	28.6	30.7±2.0
T_n.max_ (°C)	31.8	28.8	31.1	31.4	32.1	32.1	31.8	31.8	31.8	31.8	31.1	31.2	31.4±0.9
Hatch day of year	90	163	198	210	221	221	103	103	98	90	178	182	154.8±51.7
Clutch size (N)	57	45	50	70	50	63	38	47	58	53	53	72	54.7±10.0
Clutch mass (kg)	6.61	4.64	4.52	7.6	5.58	7.21	4.45	5.65	6.7	6.12	6.06	8.09	6.1±1.2
Mean egg mass (g)	115.9	103.2	90.3	108.6	111.5	114.4	117.2	120.2	115.5	115.5	114.4	112.4	111.6±8.0
Mean egg length (mm)	78.3	76.8	71.5	79.3	78.8	78.9	80.6	82.4	80.8	81.4	77.9	75.8	78.5±2.9
Mean egg width (mm)	50.3	48.3	47.1	48.5	50.2	50.2	50.2	49.6	50.2	49.9	50.7	48.9	49.5±1.1
Infertile (N)	0	2	2	5	2	4	1	9	14	15	0	0	4.5±5.3
Dead before collection (N)	1	16	27	17	7	0	0	0	2	0	0	0	5.8±9.1
Dead during incubation (N)	3	3	0	4	4	4	7	10	4	3	4	1	3.9±2.6
Total dead before hatching (N)	4	19	27	21	11	4	7	10	6	3	4	1	9.8±8.3
Live remaining (LR) eggs (N)	53	24	21	44	37	55	30	28	38	35	49	71	40.4±14.6
Abnormal hatchlings (N)	0	0	0	0	0	0	0	0	0	0	0	1	0.10±0.3
Abundant yolk hatchlings (N)	0	0	0	1	3	2	0	0	0	0	0	0	0.50±1.0
External yolk hatchlings (N)	4	1	2	1	5	0	0	4	4	4	0	0	2.1±2.0
Normal hatchlings (NH)	49	23	19	42	29	53	30	24	34	31	49	70	37.8±15.1
No. NH used in study	39	19	15	30	15	37	20	17	25	19	25	39	(25.0±9.2)
NH of LR eggs (%)	92.5	95.8	90.5	95.5	78.4	96.4	100	85.7	89.5	88.6	100	98.6	92.6±6.5
Seasonal T_min_(°C)	23.6	17.1	18.7	18.6	19.8	19.8	21.8	21.8	22.7	23.6	19.7	19.3	20.5±0.61
Seasonal T_max_ (°C)	31.8	28.8	31.1	31.4	32.1	32.1	31.8	31.8	31.8	31.8	31.1	31.2	31.4±0.26

“30°C days” is the age an embryo would be had it been incubated at 30°C [47] [48] [49]. Spot nest temperature (T_n_) was measured at the time of collection whereas mean (T_n.mean_) and maximum (T_n.max_) nest temperatures are crude estimates (Webb et al. 1987a,b,c). Lay and hatch days: January 1  =  day 1. Hatchling sizes were measured 4 days after hatching, before feeding started. The mean seasonal maximum (T_max_: 1300 h) and minimum (T_min_: 0900 h) air temperatures during the 21 days of raising for the hatchlings from each clutch (Australian Bureau of Meteorology: Darwin airport). A  =  wild nests from the Adelaide River mainstream; BP/CB  =  captive bred; M  =  wild nest from Melacca swamp on Adelaide River floodplain.

### Size

Mean EM was highly clutch specific (Table 1), which in turn affected BM_4d_ which is comprised of hatchling tissue plus the internalised residual yolk mass. Overall, there was a strong positive linear relationship between mean EM and EL of each clutch and BM_4d_ but not with SVL_4d_ (Table 2). However, mean clutch EM differed significantly between seasons (Table 2), with clutches of larger eggs laid earlier in the year (EM early  = 116.86±3.05 g; late  = 107.83±2.58 g).

**Table 2:** Relationships between egg length, egg mass, size and growth at 4, 24 and 90 days of age, and % afflicted by Failure to thrive syndrome.

	To predict	From	Formulae
**Size and Growth**			
	BM_4d_	EM	BM_4d_ = 11.677+0.541EM +2.05g; R^2^ = 0.83; *F* = 48.74; *P*<0.0001
		EL	BM_4d_ = −41.856+1.445EL+2.25g; R^2^ = 0.79; *F* = 38.78; *P*<0.0001
	SVL_4d_	EM	R^2^ = 0.10; *F* = 1.13; *P* = 0.310
		EL	R^2^ = 0.07; *F* = 0.78; *P* = 0.400
	SVL_4d_	BM_4d_ (<70g)	R^2^ = 0.003; *F* = 0.242; *P* = 0.620
		BM_4d_ (>70g)	SVL_4d_ = 76.775+0.915BM_4d_ +2.85 mm; R^2^ = 0.44; *F* = 164.28; *P*<0.0001
	SVL_24d_	BM_24d_	SVL_24d_ = −85.632+1.290BM_24d_ – (0.00497BM_24d_)^2^ ±4.13 mm; R^2^ = 0.60; *F* = 449.5; *P*<0.0001
	SVL_90d_	BM_90d_	SVL_90d_ = 147.611+0.240BM_90d_ ±8.82 mm; R^2^ = 0.94; *F* = 3865.0; *P*<0.0001
	BM_24d_	BM_4d_	R^2^ = 0.01; *F* = 3.14; *P* = 0.081
	BM_90d_	BM_4d_	R^2^ = 0.01; *F* = 2.16; *P* = 0.143
	BM_90d_	BM_24d_	BM_90d_ = −214.090+4.179BM_24d_ - 0.0252(BM_24d_-89.34)^2^ +48.69g; R^2^ = 0.62; *F* = 241.11; *P*<0.0001
	SVL_90d_	SVL_24d_	SVL_90d_ = −144.76+2.057SVL_24d_ ±16.29 mm; R^2^ = 0.44; *F* = 234.41; *P*<0.0001
	G_BM24-90d_	BM_4d_	R^2^ = 0.01; *F* = 2.03; *P* = 0.161
	G_BM4-24d_	BM_4d_	G_BM4-24d_ = 65.470 - 0.669BM_4d_ ±15.70 g; R^2^ = 0.04; *F* = 12.81; *P* = 0.0004
	G_BM24-90d_	G_BM4-24d_	G_BM24-90d_ = 19.146 + 3.147G_BM4-24d_ - 0.0377(G_BM4-24d_ -17.21) ± 50.57g; R^2^ = 0.44; *F* = 114.38; *P*<0.0001
**FTT affliction**			
	%FTT	G_BM4-24d_	%FTT = 63.9 - 7.79 G_BM4-24d_ +10.52; R^2^ = 0.94; *P = *0.0013
		BC_24d_	%FTT = 411.8-759.12 BC_24d_ +8.06; R^2^ = 0.93; *P*<0.0001
		BM_24d_	%FTT = 446.4-5.35 BM_24d_ +6.86; R^2^ = 0.96; *P = *0.0005

**Figure 1:**
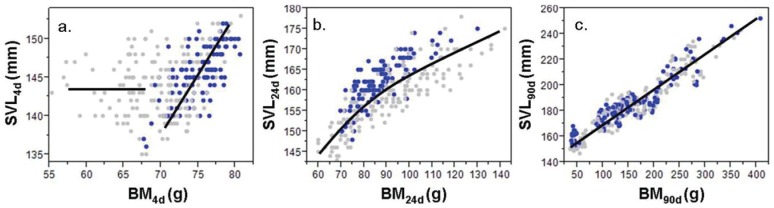
Relationship between BM and SVL of hatchling *C. porosus* (N = 300) at different ages. Relationship at a) 4d, b) 24d, and c) 90d for hatchlings born early (N = 120; blue) and late (N = 180; grey) in the year. BM_90d_ was predicted from the size-age relationship for each individual at 4, 24 and 70 to 194 days.

Overall mean BM_4d_ of *C. porosus* (N = 300) was 72.1±4.9 g (55.4–80.8 g), SVL_4d_ was 144.5±3.8 mm (135–153 mm), and BC_4d_ was 0.50±0.03 g/mm (0.39–0.50 g/mm). However, clutches of eggs laid early in the year produced hatchlings with significantly larger BM_4d_ (75.09±0.38 g) than those produced later in the year (70.15±0.31 g; Table 3). However, this was not the case with SVL at 4 days. To examine the relationship between SVL_4d_ and BM_4d_ the data set was subdivided into <70 g (N = 86) and >70 g (N = 214) BM_4d_. There was no relationship between SVL_4d_ and BM_4d_ for hatchlings with a BM_4d_ <70 g, while for hatchlings with BM_4d_ >70 g, the relationship was linear (Table 2; Fig. 1a).

**Table 3:** Seasonal differences in size, growth and %FTT between clutches laid early in the year (16–24 January 2011; 5 clutches) and clutches laid late in the year (29 March-22 May 2011; 7 clutches) using PERMANOVA with d.f. as 1 and 10.06.

Early vs late clutches	Pseudo-F	*P*(Perm*)*
EM	5.11	0.04
BM_4d_	8.09	0.02
SVL_4d_	1.70	0.23
BM_24d_	0.01	0.76
SVL_24d_	4.64	0.06
BM_90d_	0.79	0.43
SVL_90d_	1.06	0.33
G_SVL4-24d_	1.30	0.27
G_BM4-24d_	2.50	0.14
G_SVL24-90d_	0.11	0.77
G_BM24-90d_	1.25	0.33
%FTT	1.71	0.22

At 24 days of age, the overall mean BM_24d_ of *C. porosus* (N = 300) was 89.3±15.8 g (60–142 g), SVL_24d_ was 159.6±7.0 mm (143–178 mm), and BC_24d_ was 0.55±0.08 g/mm (0.41–0.80 g/mm). Clutches of hatchlings born later in the season were not significantly larger at 24 days than those born early in the year (Table 3). In contrast to the highly variable relationship between SVL and BM at 4 days, there was a much stronger relationship between BM and SVL at 24 days (Table 2; Fig. 1b).

Based on predictions at 90 days of age (N = 300), mean BM_90d_ was 162.7±75.9 g (37–409 g), SVL_90d_ was 187.7±24.8 mm (147–250 mm), and BC_90d_ was 0.80±0.33 mm (range 0.25 to 1.62 g/mm). There were no significant seasonal differences in SVL_90d_ or BM_90d_ (Table 3). The relationship between SVL and BM at 90 days of age was strongly linear (Table 2; Fig. 1c).

### Growth

Given the relatively uniform size of hatchling BM at 4 days (SD of BM_4d_  = ±4.9 g) the individual variation in size by 24 days (SD of BM_24d_  =  ±15.8 g) and 90 days (SD of BM_90d_  =  ±78.6 g) was extreme and was reflected in BC. Mean G_BM4-24d_ was 17.2±16.0 g but the range (−6.9 to 70.1 g) was already extreme with some individuals increasing by 70 g (+97.5%BM_4d_) while others had lost 7 g (−9.3%BM_4d_). Mean G_SVL4-24d_ was 15.4±6.9 mm (range 1 to 30 mm). Mean G_BM24-90d_ (63.7±67.1 g; range −40.2–278.6 g) and G_SVL24-90d_ (24.2±17.91 mm; range −3 to 77) both increased substantially relative to the 4–24 day period, but variation remained extreme. There were no seasonal differences in SVL or BM growth (Table 3).

**Figure 2:**
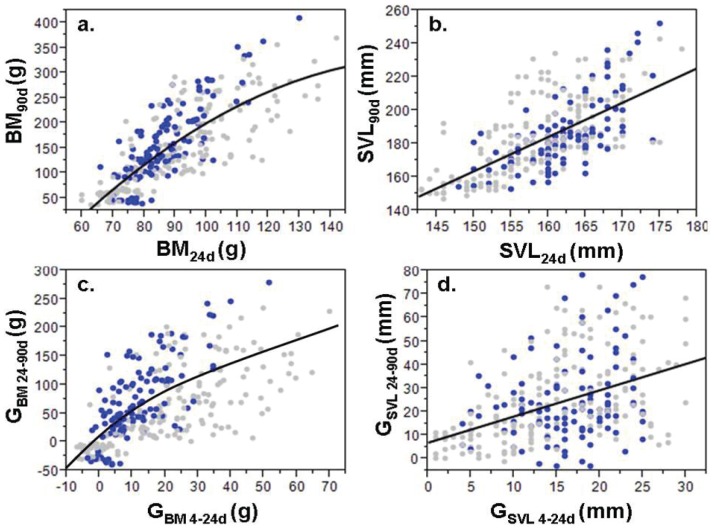
Relationship between size and growth of hatchling *C. porosus* (N = 300) at different ages. Relationship at 24 and 90 days: a) BM_24d_ and BM_90d_, b) SVL_24d_ and SVL_90d_, c) G_BM4-24d_ and G_BM24-90d_, and d) G_SVL4-24d_ and G_SVL24-90d_ for hatchlings born early (N = 120; blue) and late (N = 180; grey) in the year.

There was no significant relationship between BM_4d_ and either BM_24d_, BM_90d_, or G_BM24-90d_ (Table 2). However, BM_4d_ did have a significant but highly variable relationship with G_BM4-24d_ (Table 2), with higher growth among the smallest hatchlings born. Growth trajectories in BM and SVL established within the first 24 days were largely continued up to 90 days (Fig. 2; Table 2). A high proportion of individuals with the lowest G_BM4-24d_ and G_SVL4-24d_ and smallest BM_24d_ and SVL_24d_ failed to recover by 90 days (Fig. 2).

### Survival - FTT affliction

All animals which died during and after the study (<300 days post-hatching) were recorded. Of these, 55 (72% of mortalities) were seriously afflicted by FTT, did not respond to efforts to stimulate feeding, and died or were euthanized [31 (56.4%) at 70–100 days; 15 (27.3%) at 100–130 days; 9 (16.4%) at 130–202 days]. The remainder included animals (N = 17) that were otherwise healthy that died for other reasons between 90 and 300 days post-hatching. The proportion of individuals that died from FTT was not significantly different between seasons (Table 2).

**Figure 3:**
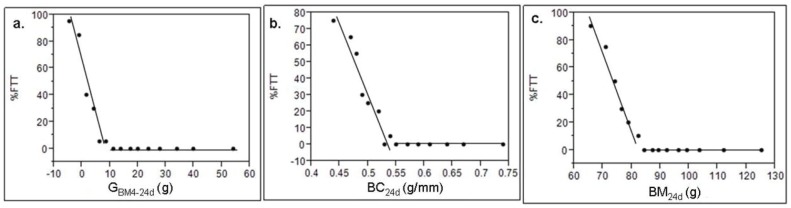
Probability of avoiding FTT and surviving to 300 days for hatchling *C. porosus* (N = 300) in relation to a) G_BM4-24d_, b) BC_24d_, and c) BM_24d_. Points are means for progress intervals (N = 20).

BM_4d_ was not significantly different between those hatchling afflicted by FTT (N = 55) and those that survived (N = 245; unequal *t*-test: *t* = −0.429; df  = 298; *P* = 0.668). However, the probability of affliction with FTT was clearly indicated within the first 24 days, by the extent of growth in body mass (Table 2; Fig. 3a), body condition (Table 2; Fig. 3b), and body mass (Table 2; Fig. 3c). No affliction by FTT (0%FTT) was detected in animals that grew more than 8.2 g, achieved a BC_24d_ of 0.55 g/mm SVL or a BM_24d_ of 81.7 g in the first 24 days post-hatching. However, there were a total of 55 hatchlings that grew less than 8.2 g after 24 days and survived. These hatchlings grew significantly less (44.43±4.59 g; Welch's t-test: *t* = 7.49; df  = 243; *P*<0.0001) between 24 and 90 days and were significantly smaller at 90 days (122.48±4.67 g; Welch's t-test: *t* = 9.82; df  = 243; *P*<0.0001) compared with other hatchlings (G_BM24-90d_ = 92.03±4.40; BM_90d_ = 189.28±4.95).

### Clutch effects

Among the non-FTT individuals (N = 245), clutch had a significant effect on BM_4d_, G_BM4-24d_, and BM_24d_ (Table 4). As growth trajectories established within the first 24 days are continued to 90 days, clutch effects were also apparent in G_BM24-90d_ and BM_90d_ (Table 4). However, if the variance due to G_BM4-24d_ is removed, no remaining clutch variation occurred in BM_24d_, G_BM24-90d_, or BM_90d_. This confirms that the clutch variation detected was mainly due to variation in G_BM4-24d._

**Table 4:** BM_4d_, BM_24d_, BM_90d_, G_BM4-24d_, G_BM24-90d_, and for non-FTT animals (N  =  245) and the percentage of FTT animals (N = 55) according to clutch.

Variable	ANOVA	Clutch ID											
		A20	A44	A70	A71	A75	A77	BP4	CB5	M28	M43	M55	M62
													
BM_4d_ (g)	R^2^ = 0.68; *F* = 45.14; *P*<0.0001												
Mean		73.9	67.6	61	72.2	71.6	71.9	77.3	77.7	74.5	73.9	76.2	67.8
SD		3.3	1.9	3.4	2.2	3.7	3.6	1.8	2.4	2.7	2.2	2.6	3.1
BM_24d_ (g)	R^2^ = 0.24; *F* = 6.62; *P*<0.0001												
Mean		85.6	99.2	82.6	101.6	88.6	102.4	92.9	98.7	84.8	83.2	92.6	97.3
SD		7.1	16.2	10.5	17.1	3.7	18.9	13.1	12.6	10.9	5.5	8.5	13.5
BM_90d_ (g)	R^2^ = 0.23; *F* = 6.23; *P*<0.0001												
Mean		142.2	181.1	125.3	185.6	123.1	215.3	158.6	260.9	152.6	111.5	96.9	178.8
SD		56.9	73.3	70.9	64.5	18.6	86.0	88.4	61.4	60.8	53.2	54.2	53.0
G_BM4-24d_ (g)	R^2^ = 0.32; *F* = 10.01; *P*<0.0001												
Mean		11.7	31.6	21.6	29.4	16.9	30.5	15.6	20.9	10.3	9.3	16.4	29.5
SD		6.3	15.9	9.3	16.9	4.1	18.4	12.3	12.4	9.6	5.3	9	13.2
G_BM24-90d_ (g)	R^2^ = 0.23; *F* = 6.23; *P*<0.0001												
Mean		68.0	81.9	58.9	82.1	34.5	112.9	83.7	162.3	72.5	52.7	42.9	84.3
SD		43.8	61.9	62.8	59.1	18.6	74.5	68.7	50.5	49.6	34.7	42.3	41.7
													
%FTT		10.3	0	20	23.3	53.3	27	15	0	8	26.3	48	2.6

Across clutches, the mean incidence of FTT was 19.5+4.99% of hatchlings, but the range varied from 0% to 53.3%, demonstrating highly significant clutch effects (*X* ^2^ = 48.36, df  = 11, *P*<0.0001; Table 4). None of the clutch-specific variation in’FTT could be explained by the mean clutch and incubation characteristics (Table 1), although it was a relatively small sample (N = 12) and none of these variables were controlled.

## Discussion

Our results suggest that under similar experimental conditions, growth trajectories for the majority of *C. porosus* hatchlings established within the first 24 days post-hatching extend to 90 days and beyond. Similarly, individuals with a high probability of affliction by FTT up to 300 days post-hatching can be identified within the first 24 days by reduced growth. Therefore, instead of conforming to the ‘bigger is better’ hypothesis, hatchling *C. porosus* under these conditions appear to benefit from rapid early growth. However, whether this is the situation in the wild, where the environment is vastly different to that in captivity, is unknown.

While insights into the fitness of animals can be gained from both captive and wild animals, results need to be merged and assessed carefully. Growth and survival of neonate snakes (*Thamnophis sirtalis*) under captive conditions were similar to those in the field [12] [58]. Yet other animals held in captivity can experience either greater or less fitness than their wild counterparts. Species which suffer high levels of stress [59] in captivity generally appear to be less fit, and this has been reported in certain species of lemur [60] [61], dolphin [62] [63], parrot [64] [65], and raptor [66], and predictably so if their ecology and response to humans is considered [67] [59].

For hatchling *C. porosus* under captive conditions, resources such as temperature, cover and food are abundant and there is no risk of predation [55]. However, individuals are confined and forced to live at higher than natural densities, and are subject to human disturbance [55]. In the wild, the availability of resources can often be limited or can fluctuate while the threat of predation is high and hatchling *C. porosus* must contend with larger crocodiles [36] [49]. Female *C. porosus* also protect their offspring for the first few weeks and months post hatching [36] [49]. As such, differences may exist in terms of which traits (size etc) may be selected for in captivity and in the wild, and this in turn may vary according to location and habitat.

For *C. porosus*, and many other crocodilians, there may be advantages in attaining a large size rapidly, in terms of the ability to avoid predation, compete with conspecifics, survive adverse environmental conditions, and reach sexual maturity [68]. In the majority of cases, aggressive encounters between crocodilians favour the larger animal [45] [69] [70], which then enables greater access to resources and subsequently improved long-term fitness both in the wild and in captivity [68] [55]. *Crocodylus porosus* is considered the most aggressive and intolerant of conspecifics of all crocodilians [70], and agonistic behaviour begins within two days of hatching [45]. Such behaviours are known to affect growth and survival in several species of reptile [71] [72] [73] [74], and this also appears to be the case in *C. porosus* under captive conditions [55] [75] [39].

Clutch of origin and the incubation environment have been widely reported to affect post-hatching growth and survival in crocodilians [42] [76] [77] [37] [78] [39]. Therefore, we tried to quantify sources of variation within the clutch, egg and incubation variables that may have biased our results (Table 1). None explained the variation in growth or affliction with FTT. However, the results highlight the inherent complexity of potential variables that may influence growth and survival, and the importance of assumptions about the homogeneity of neonates used for such raising trials [42] [39].

That FTT can be predicted after 24 days, suggests that the first few weeks post-hatching are crucial to short-term fitness of *C. porosus* under captive conditions with survival increasing up to 90% in individuals that increased in mass by 4–7g during this period. While this has been suggested for crocodilians by previous authors [37] [55], it has never been accurately quantified for any species. Regardless of whether this is the situation in the wild, it does suggest that if early conditions are unfavourable then short-term growth and survival can be compromised. This has been found in water pythons [22] in which different rates of growth and survival occur between years based on prey abundance during the early post-hatching stage.

The occurrence of FTT among captive-raised crocodilians is widespread, although because weakened animals are vulnerable to secondary illnesses, FTT may be under-reported [41]. Regardless, *C. porosus* appear particularly prone to FTT affliction [40] [42] [39]. FTT is generally considered to result from an inadequate raising environment, although what constitutes an adequate raising environment for each species remains poorly understood and may be more species-specific than previously realised. For example, *Alligator mississippiensis* have substantially higher rates of growth and survival to one year of age when raised under identical conditions to *C. porosus* [82] [73]. Hatchling *A. mississippiensis* are reported to initiate feeding more rapidly and on a wider range of food types, and as a species are considered far more tolerant of conspecifics with no or little aggression reported among juveniles in captivity [79] [68] [55] [70]. Therefore, it is possible that the current approach to raising *C. porosus* in captivity, which was originally based on the model used for *A. mississippiensis* [79] [68], may be inadequate.

The extent to which FTT occurs in wild populations of *C. porosus* is not well understood, and would be difficult to quantify due to (presumably) an increased vulnerability of these weakened animals to predation. However, while emaciated or malnourished hatchling *C. acutus* [80], *A. mississippiensis*, and *C. johnstoni* (M. Brien pers. observation) have been observed in the wild on a number of occasions, hatchling *C. porosus* in an emaciated state have rarely been encountered in the wild [81] [82]. Hence, FTT may not occur in wild *C. porosus* at anything like the rates reported in captivity [39]. If so, genetic predispositions to FTT, which could be complicated by multiple paternity [83], may be a response to threats that can be avoided by appropriate behaviour in the wild, but not in captivity.

Factors that affect survival rates in hatchling *C. porosus* under captive conditions have clear implications on future growth and survival. However, it is not really clear that enhanced growth trajectories in the hatchling stage, forewarned in the first 24 days, will ultimately influence “fitness” of individuals in the long-term. It is unlikely that measured variation in growth within a time scale of 24 days will ultimately be correlated with variation in reproductive performance after a time scale of up to 20+ years [38] [84]. This is because a completely different suite of factors dictate progress and outcomes during this time [85].

Variation in the survival and growth rates of *C. porosus* hatchlings in controlled environments are intimately connected to each other, particularly through FTT. Absolute growth, independent of hatchling size, is perhaps the best index of individual performance, which has implications for survival within captive environments, where the goal is often to enhance both survival and the early attainment of large juvenile size. However, it is important to realise that some hatchlings can recover from poor growth rates within the first 24 days (<8.2 g). Identifying and understanding the causes of FTT among hatchling crocodilians is essential for improving conservation and management programs aimed at raising crocodilians that are threatened or endangered for purposes such as head starting, in which individuals are released back to the wild at a size that ensures greater survival.

## Supporting Information

Data S1 **Individual size and growth data at 4 days, 24 days and predicted at 90 days for 300 hatchlings.**
(XLS)
